# Molecular determinants analysis and co receptor tropism prediction of V3 loop of HIV-1 “C” clade sequences isolated from India using insilico approach

**DOI:** 10.1186/1471-2334-12-S1-O18

**Published:** 2012-05-04

**Authors:** H Jemmy Christy, D Alex Anand, Renuka Pasupuleti

**Affiliations:** 1 Sathyabama University, Chennai, India

## Background

The V3 loop of the *env* gene in HIV-C type is considered as a major viral determinant for coreceptor specificity. The tip motif of v3 loop is known for antibody neutralization, so sequence variation in this motif have an impact on virus infectivity and disease progression. So analysis of genetic diversity in the V3 loop Tip motif help us to improve the CCR5 antagonist’s development as well as vaccine target.

## Methods

HIV-1 “C” type *env* sequences of Indian isolates were retrieved from HIV sequence database. Coreceptor Tropism prediction by different web-based interpretation system like (WebPSSM, Geno2pheno, (ds) Kernel, WetCat) was performed. The diversity of V3 loop tip motif, glycosylation motifs were analyzed using N-Glycosite. Relative frequencies of each amino acid in v3 loop were determined using Web Logo.

## Results

In this study, lower numbers of positive charges of v3 loop, in the range of 4 to 5, reveals the prevalence of R5 tropism. Thus majority of the V3 sequences of HIV-1 Indian isolates were predicted as R5-tropism and we identified few mutational prevalence like for R5-predicted viruses -E25D, Y21F and for X4-predicted viruses E25KRQ, I12, H34Y. Overall eight different tetrameric tip motifs [GPGQ, GPGR, GPAQ, GPRR, GPGL, RPRQ, EPGQ, and GSGQ] were identified.

## Conclusion

High prevalence of R5 tropism, higher no of conserved motif regions in the V3 sequence among HIV strains in India reveals the need of potential CCR5 antagonists. GPGQ and GPGR is highly conserved in all the sequences and this pattern could be an ideal target for AIDS therapy.

